# Extended Approaches to the Maxillary Sinus are not Associated With an Increased Risk of Empty Nose Syndrome

**DOI:** 10.1002/alr.23513

**Published:** 2025-01-06

**Authors:** Oloruntobi Rotimi, Isabelle Williams, Catherine Rennie, Saleh Okhovat, Rishi Sharma, Peter‐John Wormald, Neil C.‐W. Tan

**Affiliations:** ^1^ ENT Department University College Hospital London UK; ^2^ ENT Department Evelina London Children's Hospital London UK; ^3^ ENT Department Charing Cross Hospital Imperial College NHS Trust London UK; ^4^ ENT Department Glasgow Royal Infirmary and Queen Elizabeth University Hospital Glasgow UK; ^5^ ENT Department Addenbrooke's Hospital Cambridge UK; ^6^ Department of Surgery – Otolaryngology Head and Neck Surgery Adelaide Universities The Queen Elizabeth Hospital Woodville South Australia Australia; ^7^ ENT Department Royal Cornwall Hospital Truro UK; ^8^ University of Exeter Medical School Royal Cornwall Hospital Truro UK

**Keywords:** empty nose syndrome, endoscopic sinus surgery, maxillectomy, patient reported outcome measures

## 1 Introduction

Empty nose syndrome (ENS) is a rare complication of sinonasal surgery which is characterized by the paradoxical sensation of nasal obstruction despite a widely patent nasal cavity [1, 2]. ENS is a debilitating condition that has negative physical and psychological impacts on quality of life [3]. Several studies found no significant association between turbinate resection and the development of ENS [4, 5]. Notably, there is a paucity of data examining the association between more extensive sinonasal surgery and ENS [6]. The study aims to assess the association between extended approach to the maxillary sinus procedures (EAMS) and ENS.

**Summary**

- Extended approaches to the maxillary sinus are not associated with an increased risk of Empty Nose Syndrome.
- No difference in the ENS6Q scores between turbinate sparing and turbinate sacrificing procedures.

## 2 Methods

A retrospective cohort compared the association of ENS in adult patients who have undergone EAMS against adult patients who underwent endoscopic sinus surgery (ESS) in four tertiary centers across the United Kingdom. Consecutive patients who underwent EAMS for benign sinonasal disease were included. Exclusion criteria included: pediatric patients, malignant disease, incomplete data, and previous sinonasal surgery. Control participants underwent ESS for chronic rhinosinusitis (CRS). EAMS and ESS groups were matched to minimize bias due to demographics. EAMS included radical medial maxillectomy (RMM), modified medial maxillectomy (MMM), or prelacrimal approach (PLA) [7] (Appendix S2). ESS was defined as full house ESS without formation of a mega‐antrostomy. Outcome measures were obtained during outpatient reviews. The primary measure was the 6—item empty nose syndrome questionnaire score (ENS6Q).

Mann–Whitney *U* test, Fischer exact test, and descriptive characteristics were conducted using IBM SPSS (Version 29.0.2.0). All tests were two‐tailed (significance threshold of 0.05).

## 3 Results

**FIGURE 1:**
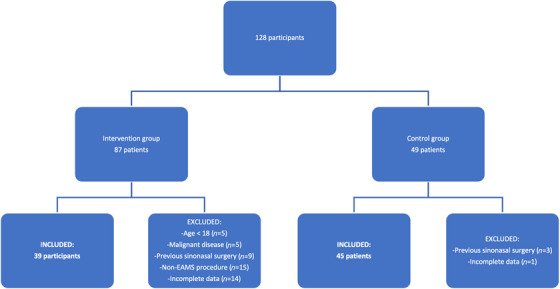
Flow diagram of study participants.

A total of 136 patients were screened for eligibility. Out of them, 84 patients were included in this study. The rationale for the excluded patients are displayed in Figure 1.

A total of 39 patients underwent EAMS (23 RMM; 16 MMM/PLA) and 45 patients underwent ESS. The EAMS and ESS groups were matched for gender and age as shown in Table 1.

**TABLE 1:** Patient characteristics (SD, standard deviation).

	EAMS (intervention)	ESS (control)	*p* Value
Gender	22 Male 17 Female	26 Male 19 Female	*p* > 0.05
Median age ± SD	60 ± 14.4	56 ± 16.5	*p* > 0.05

87% of patients in the EAMS group (*n* = 34) underwent surgery for inverted papilloma with no evidence of atypia or malignant transformation on histology. The remaining five patients underwent surgery for benign vascular lesions. The patients in the ESS group underwent surgery for refractory CRS to appropriate medical therapy.

There was no statistically significant difference in mean ENS6Q scores in patients who underwent EAMS compared with ESS (2.77 ± 3.94 vs. 3.27 ± 4.29, *p* = 0.65). There was no statistically significant difference in ENS6Q scores in patients who underwent turbinate preserving procedures (e.g., MMM/PLA) compared with turbinate sacrificing procedures (e.g., RMM) (2.57 ± 3.46 vs. 3.06 ± 4.64, *p* = 0.81). There was a statistically significant difference in nasal suffocation subscore between the EAMS group and ESS group (nasal suffocation, *p* = 0.009). There were no statistically significant differences in the remaining ENS6Q subscores between the EAMS group and the ESS group, as shown in Table 2.

**TABLE 2:** ENS6Q subscores within the EAMS and ESS cohorts (figures quoted as mean ± standard deviation).

ENS6Q subscore	EAMS group	ESS group	*p* Value
Nasal dryness	0.62 ± 1.09	0.64 ± 1.07	*p* >0.05
Sensation of poor nasal airflow	0.69 ± 1.32	1.11 ± 1.42	*p* > 0.05
**Nasal suffocation** ^a^	**0.13** ± **0.66**	**0.58** ± **1.16**	***p* >0.05**
Sensation of Nasal openness	0.15 ± 0.71	0.24 ± 0.74	*p* < 0.05
Nasal crusting	0.97 ± 1.22	0.24 ± 0.77	*p* > 0.05
Nasal burning	0.15 ± 0.71	0.44 ± 1.16	*p* >0.05

^a^ Statistically significant.

## 4 Discussion

This study was unable to identify any significant association between EAMS procedures and increased total ENS6Q scores. There were also no significant differences in ENS6Q between patients undergoing EAMS procedures and patients undergoing ESS. This suggests there is no additional risk of developing ENS when comparing patients undergoing EAMS with those undergoing ESS. There was no evidence for an additional risk of developing ENS when comparing turbinate sacrificing surgery turbinate sparing procedures, which is consistent with the findings in existing literature [4, 5]. Patients undergoing EAMS experienced less nasal suffocation than those undergoing ESS. Although this was statistically significant, this is unlikely to be clinically significant as the subscores were less than 1.

The evidence refuting altered turbinate morphology as a cause of ENS is mounting and suggests other mechanisms may be present as predisposing factors in the pathogenesis of this condition, that is, altered nasal airflow dynamics, disrupted nasal mucosal function, and impaired neurosensory mechanisms [8, 9]. Li et al. [9] assessed nasal airflow using computational fluid dynamics in ENS patients and found that nasal airflow was disproportionately directed to the middle meatus compared with controls (56.2 ± 14.7% of total flow). Nasal mucosa contains receptors that detect cool airflow to trigger nasal heating and humidification [8]. Disruption of nasal humidification impacts on the laminar nasal airflow and encourages crust formation which can subsequently alter neurosensory mechanisms [8]. These preexisting factors may predispose certain individuals to developing ENS following turbinate resection.

This is the first study of its kind to explore the association between ENS and EAMS procedures, laying the groundwork for further experimental research. The authors adopted a multicenter approach which enhanced the external validity of the findings and minimized recruitment bias, while the matching of patient demographics helped to minimize confounding variables.

Limitations include a small sample size and the retrospective design of the study. Furthermore, the observational nature of this study does not demonstrate temporality therefore causation cannot be easily inferred. Last, the control participants were obtained from a single center, limiting generalizability (Appendix S1).

The authors recommend further research with diagnostic studies to reliably identify predisposing factors for ENS. This would aid clinical decision‐making in terms of patient selection preoperatively and the extent of resection intraoperatively, ultimately reducing the incidence of patients with post‐operative ENS symptoms.

## Conflicts of Interest

The authors declare no conflicts of interest.

## Supporting information



Supporting Information

Supporting Information
