# Supplementary material for: Extended Approaches to the Maxillary Sinus are not Associated With an Increased Risk of Empty Nose Syndrome
Source: Int Forum Allergy Rhinol. 2025 Jan 6;15(4):445–7. doi: 10.1002/alr.23513 (PMC11970443; doi:10.1002/alr.23513)
Supplement: Supplementary file 1 — Supporting Information [file ALR-15-445-s002.docx]

**Appendix 1**

| Patient | Dryness | Crusting | Nasal suffocation | Nasal burning | Nasal openness | Impaired air sensation | Total |
| --- | --- | --- | --- | --- | --- | --- | --- |
| EAMS1 | 0 | 0 | 0 | 0 | 0 | 0 | 0 |
| EAMS2 | 0 | 0 | 0 | 0 | 0 | 0 | 0 |
| EAMS3 | 0 | 0 | 0 | 0 | 0 | 0 | 0 |
| EAMS4 | 0 | 0 | 0 | 0 | 1 | 0 | 1 |
| EAMS5 | 2 | 4 | 0 | 0 | 3 | 4 | 13 |
| EAMS6 | 0 | 0 | 0 | 0 | 0 | 0 | 0 |
| EAMS7 | 0 | 0 | 0 | 0 | 0 | 0 | 0 |
| EAMS8 | 2 | 0 | 0 | 0 | 1 | 0 | 3 |
| EAMS9 | 0 | 0 | 0 | 0 | 0 | 0 | 0 |
| EAMS10 | 2 | 2 | 0 | 0 | 0 | 0 | 4 |
| EAMS11 | 2 | 0 | 0 | 0 | 2 | 0 | 4 |
| EAMS12 | 0 | 0 | 0 | 0 | 0 | 0 | 0 |
| EAMS13 | 0 | 3 | 0 | 0 | 2 | 0 | 5 |
| EAMS14 | 0 | 0 | 0 | 0 | 1 | 0 | 1 |
| EAMS15 | 2 | 4 | 0 | 0 | 3 | 0 | 9 |
| EAMS16 | 1 | 1 | 0 | 2 | 2 | 0 | 6 |
| EAMS17 | 0 | 0 | 0 | 0 | 0 | 0 | 0 |
| EAMS18 | 0 | 0 | 0 | 0 | 0 | 0 | 0 |
| EAMS19 | 0 | 0 | 0 | 0 | 0 | 0 | 0 |
| EAMS20 | 5 | 4 | 4 | 0 | 5 | 0 | 18 |
| EAMS21 | 0 | 0 | 0 | 0 | 2 | 0 | 2 |
| EAMS22 | 1 | 1 | 0 | 0 | 0 | 0 | 2 |
| EAMS23 | 1 | 3 | 0 | 0 | 3 | 0 | 7 |
| EAMS24 | 0 | 0 | 0 | 0 | 0 | 0 | 0 |
| EAMS25 | 1 | 3 | 1 | 0 | 0 | 2 | 7 |
| EAMS26 | 0 | 0 | 0 | 0 | 0 | 0 | 0 |
| EAMS27 | 0 | 0 | 0 | 0 | 2 | 0 | 2 |
| EAMS28 | 1 | 0 | 0 | 0 | 0 | 0 | 1 |
| EAMS29 | 0 | 0 | 0 | 0 | 0 | 0 | 0 |
| EAMS30 | 1 | 0 | 0 | 0 | 1 | 0 | 2 |
| EAMS31 | 0 | 2 | 0 | 0 | 1 | 0 | 5 |
| EAMS32 | 0 | 0 | 0 | 4 | 2 | 0 | 6 |
| EAMS33 | 0 | 0 | 0 | 0 | 0 | 0 | 0 |
| EAMS34 | 0 | 0 | 0 | 0 | 2 | 0 | 2 |
| EAMS35 | 0 | 0 | 0 | 0 | 0 | 0 | 0 |
| EAMS36 | 3 | 0 | 0 | 0 | 2 | 0 | 5 |
| EAMS37 | 0 | 0 | 0 | 0 | 0 | 0 | 0 |
| EAMS38 | 0 | 0 | 0 | 0 | 1 | 0 | 1 |
| EAMS39 | 0 | 0 | 0 | 0 | 2 | 0 | 2 |

| Patient | Dryness | Crusting | Nasal suffocation | Nasal burning | Nasal openness | Impaired air sensation | Total |
| --- | --- | --- | --- | --- | --- | --- | --- |
| ESS1 | 1 | 1 | 0 | 0 | 0 | 0 | 2 |
| ESS2 | 2 | 1 | 0 | 0 | 0 | 2 | 5 |
| ESS3 | 0 | 3 | 4 | 4 | 0 | 0 | 11 |
| ESS4 | 0 | 2 | 2 | 0 | 0 | 0 | 4 |
| ESS5 | 0 | 3 | 0 | 0 | 0 | 0 | 3 |
| ESS6 | 2 | 2 | 0 | 0 | 1 | 0 | 5 |
| ESS7 | 3 | 3 | 4 | 2 | 1 | 5 | 18 |
| ESS8 | 2 | 1 | 0 | 0 | 0 | 3 | 6 |
| ESS9 | 2 | 4 | 0 | 0 | 0 | 3 | 9 |
| ESS10 | 0 | 0 | 0 | 0 | 0 | 0 | 0 |
| ESS11 | 0 | 3 | 1 | 2 | 0 | 0 | 6 |
| ESS12 | 1 | 3 | 0 | 0 | 0 | 0 | 4 |
| ESS13 | 2 | 2 | 0 | 0 | 0 | 0 | 4 |
| ESS14 | 0 | 0 | 0 | 0 | 0 | 0 | 0 |
| ESS15 | 1 | 1 | 0 | 1 | 3 | 2 | 8 |
| ESS16 | 0 | 3 | 0 | 0 | 0 | 0 | 3 |
| ESS17 | 0 | 3 | 0 | 0 | 0 | 0 | 3 |
| ESS18 | 0 | 0 | 0 | 1 | 0 | 0 | 1 |
| ESS19 | 0 | 0 | 0 | 0 | 0 | 0 | 0 |
| ESS20 | 0 | 0 | 1 | 0 | 0 | 0 | 1 |
| ESS21 | 1 | 5 | 1 | 0 | 0 | 0 | 7 |
| ESS22 | 0 | 1 | 0 | 0 | 0 | 0 | 1 |
| ESS23 | 0 | 0 | 0 | 0 | 0 | 0 | 0 |
| ESS24 | 4 | 4 | 4 | 0 | 0 | 0 | 12 |
| ESS25 | 0 | 1 | 0 | 0 | 0 | 0 | 1 |
| ESS26 | 0 | 0 | 0 | 0 | 0 | 0 | 0 |
| ESS27 | 0 | 0 | 0 | 0 | 0 | 0 | 0 |
| ESS28 | 0 | 0 | 0 | 0 | 0 | 0 | 0 |
| ESS29 | 0 | 0 | 0 | 0 | 0 | 0 | 0 |
| ESS30 | 0 | 0 | 0 | 0 | 0 | 0 | 0 |
| ESS31 | 0 | 0 | 0 | 0 | 0 | 0 | 0 |
| ESS32 | 1 | 1 | 0 | 1 | 3 | 1 | 7 |
| ESS33 | 0 | 0 | 0 | 0 | 0 | 0 | 0 |
| ESS34 | 0 | 0 | 1 | 0 | 0 | 0 | 1 |
| ESS35 | 0 | 0 | 0 | 0 | 0 | 0 | 0 |
| ESS36 | 1 | 0 | 0 | 0 | 0 | 0 | 1 |
| ESS37 | 0 | 0 | 0 | 0 | 0 | 0 | 0 |
| ESS38 | 0 | 0 | 0 | 0 | 0 | 0 | 0 |
| ESS39 | 0 | 0 | 0 | 0 | 0 | 0 | 0 |
| ESS40 | 0 | 0 | 2 | 0 | 0 | 0 | 2 |
| ESS41 | 3 | 2 | 2 | 0 | 3 | 4 | 14 |
| ESS42 | 0 | 0 | 0 | 0 | 0 | 0 | 0 |
| ESS43 | 0 | 0 | 0 | 0 | 0 | 0 | 0 |
| ESS44 | 3 | 1 | 3 | 0 | 0 | 0 | 7 |
| ESS45 | 0 | 0 | 1 | 0 | 0 | 0 | 1 |
