# Supplementary material for: Extended Approaches to the Maxillary Sinus are not Associated With an Increased Risk of Empty Nose Syndrome
Source: Int Forum Allergy Rhinol. 2025 Jan 6;15(4):445–7. doi: 10.1002/alr.23513 (PMC11970443; doi:10.1002/alr.23513)
Supplement: Supplementary file 2 — Supporting Information [file ALR-15-445-s001.docx]

Appendix 2: Definitions of Extended approaches to the maxillary sinus

***Extended approaches to the Maxillary Sinus***

Extended approaches to the maxillary sinus include procedures such as:

Radical Medial Maxillectomy

Modified Medial Maxillectomy

Pre-lacrimal approach

Radical Medial Maxillectomy involves resection of the medial wall of the maxilla including the entire inferior turbinate.

Modified medial maxillectomy involves resection of the medial wall of the maxilla and the posterior half of the inferior turbinate whilst preserving the head of the inferior turbinate.

Pre-lacrimal approach involves resection of the medial wall of the maxilla anterior to the nasolacrimal duct.
